# Induced Pluripotent Stem Cells to Study Mechanisms of Laminopathies: Focus on Epigenetics

**DOI:** 10.3389/fcell.2018.00172

**Published:** 2018-12-20

**Authors:** Silvia Crasto, Elisa Di Pasquale

**Affiliations:** ^1^Institute of Genetic and Biomedical Research, National Research Council of Italy, UOS of Milan, Milan, Italy; ^2^Humanitas Research Hospital, Rozzano, Milan, Italy

**Keywords:** induced pluripotent stem cells (iPSCs), LMNA, Lamin A/C, laminopathies, cell differentiation, epigenetic regulation, chromatin architecture, gene expression regulation

## Abstract

Laminopathies are a group of rare degenerative disorders that manifest with a wide spectrum of clinical phenotypes, including both systemic multi-organ disorders, such as the Hutchinson-Gilford Progeria Syndrome (HGPS), and tissue-restricted diseases, such as Emery-Dreifuss muscular dystrophy, dilated cardiomyopathy and lipodystrophies, often overlapping. Despite their clinical heterogeneity, which remains an open question, laminopathies are commonly caused by mutations in the LMNA gene, encoding the nuclear proteins Lamin A and C. These two proteins are main components of the nuclear lamina and are involved in several biological processes. Besides the well-known structural function in the nucleus, their role in regulating chromatin organization and transcription has emerged in the last decade, supporting the hypothesis that the disruption of this layer of regulation may be mechanism underlying the disease. Indeed, recent studies that show various epigenetic defects in cells carrying LMNA mutations, such as loss of heterochromatin, changes in gene expression and chromatin remodeling, strongly support this view. However, those findings are restricted to few cell types in humans, mainly because of the limited accessibility of primary cells and the difficulties to culture them *ex-vivo*. On the other hand, animal models might fail to recapitulate phenotypic hallmarks of the disease as of humans. To fill this gap, models based on induced pluripotent stem cell (iPSCs) technology have been recently generated that allowed investigations on diverse cells types, such as mesenchymal stem cells (MSCs), vascular and smooth muscle cells and cardiomyocytes, and provided a platform for investigating mechanisms underlying the pathogenesis of laminopathies in a cell-type specific human context. Nevertheless, studies on iPSC-based models of laminopathy have expanded only in the last few years and, with the advancement of reprogramming and differentiation protocols, their number is expecting to further increase over time. This review will give an overview of models developed thus far, with a focus on the novel insights on epigenetic mechanisms underlying the disease in different human cellular contexts. Perspectives and future directions of the field will be also given, highlighting the potential of those models for preclinical studies for identifying molecular targets and their translational impact on patients' cure.

## Introduction

Laminopathies are a group of rare disorders that manifest with a wide spectrum of clinical phenotypes. In spite of their heterogeneity, mutations in LMNA gene, encoding the nuclear proteins Lamin A and C, represent the main common cause of this group of diseases (Burke and Stewart, 2006; Worman, 2012).

How can mutations in a single gene give rise to so many diverse and tissue-specific phenotypes is still one of the major unanswered questions in the field, and it is likely to be dependent on epigenetic mechanisms.

The difficulty to access specific human tissues (such as the heart) and to culture cells isolated from them *ex-vivo*, have represented a major obstacle in dissecting the mechanisms behind this group of pathologies.

Much of what we have learnt so far on the molecular and functional mechanisms of laminopathies has come from the analysis of dermal fibroblasts, skeletal muscle cells and overexpression studies in human cell lines (Goldman et al., 2004; Scaffidi and Misteli, 2006; Shumaker et al., 2006), or from the development of mouse models (Mounkes et al., 2003; Yang et al., 2005; Hernandez et al., 2010; Le Dour et al., 2017; Hamczyk et al., 2018). However, besides the importance of these models in gaining knowledge on laminopathies, they may deviate from the “natural” human condition in terms of physiology, genetic landscape and chromatin signature.

iPSC technology represents an ideal approach to overcome these limitations, allowing generation of any cell type of the body through specific differentiation protocols (Takahashi and Yamanaka, 2016); indeed, tissue-specific models of laminopathies have recently been generated from iPSCs, that recapitulate traits of the disease *in vitro* (Liu et al., 2011a; Zhang et al., 2011, 2014; Siu et al., 2012; Xiong et al., 2013). Results obtained so far already contributed to clarify some functional and molecular mechanisms of the disease in the human context, and those that will emerge from future studies will surely bring to light novel mechanistic insights into their pathogenesis. We can expect that these new findings will set the stage for application of iPSC-based models to pharmacological testing in tissue-specific contexts (Blondel et al., 2014, 2016; Lee et al., 2017), making the technology available to patients.

This review focuses on the iPSC technology applied to laminopathies, with the specific intent to illustrate the complexity of this field by describing findings related to available cellular models. In particular, we will give a special emphasis to the epigenetic role of Lamin A/C, highlighting the effects of Lamin A/C on gene transcription and chromatin remodeling in cells of different derivation: we will describe how disruption of Lamin A/C-mediated epigenetic regulation may be a mechanism of disease in different cellular contexts and represent a potential target for development of “specific” drugs.

## LMNA, Lamin A/C, and Laminopathies

Lamins are nuclear proteins, classified as type V intermediate filaments (IF): these proteins assemble in a hierarchical fashion to form isoform-specific dense filamentous meshworks which interact with a large number of binding partners to constitute the nuclear lamina, and provide structural support to the nucleus (de Leeuw et al., 2018). In addition to this structural role, lamins are also involved in other cellular processes, such as chromatin organization and DNA replication and repair (Burke and Stewart, 2013; de Leeuw et al., 2018). The spatial architecture of chromosomes and the folding of the chromatin fiber are known to be important for gene regulation and genome maintenance (Misteli and Soutoglou, 2009; Kind and van Steensel, 2010).

In terms of protein structure, lamins share similar domains with other IF proteins (i.e., desmin and vimentin, IF type III, keratins, type I and II), but the folding of the full-length protein has not yet been reported, and only subdomains of lamins have been crystallized (Ruan et al., 2012).

Recently, Turgay et al. were able to resolve the filamentous meshwork organization and to acquire structural details of lamin filaments in mammalian cells, using cryo-electron tomography (cryo-ET) (Turgay et al., 2017). However, due to a resolution limit, it was impossible to distinguish A-type from B-type lamins.

In mammalian cells, four lamin isoforms are predominantly present and are grouped into A-type (A and C) or B-type (B1 and B2). Originally, these proteins have been classified based on their isoelectric point: A-type Lamins, with a near-neutral isoelectric point (Gerace and Blobel, 1980), and B-type Lamins with acidic isoelectric point (Krohne and Benavente, 1986). Furthermore, A-type lamins can be distinguished from B-type ones depending on their primary sequence and their tissue specific expression. In fact, while B-type lamins are ubiquitously expressed, those of A-type are mostly expressed in differentiated cells and are absent or expressed in reduced quantities in early embryos, pluripotent stem cells and certain neurons (Worman and Bonne, 2007; Adam and Goldman, 2012).

At the genomic level, B-type lamins (B1 and B2) are transcribed from two distinct genes (*LMNB1* and *LMNB2*), whereas Lamin A and C proteins are encoded by a single gene (*LMNA*) then separated through an alternative RNA splicing event within exon 10. As a consequence, Lamin C is identical to Lamin A up to codon 566, after which it lacks part of exon 10 as well as exons 11 and 12, but possesses five unique basic amino acid residues at its C-terminus (Lin and Worman, 1993). Lamin A is instead translated as prelamin A that undergoes a multistep processing via post-translational modifications to lead to Lamin A; specifically, farnesylation at the CAAX motif, carboxymethylation, and endoproteolysis of the last 18 amino acids by ZMPSTE24 sequentially occur and mediate the production of mature Lamin A (Young et al., 2005; Rusinol and Sinensky, 2006). In the Hutchinson–Gilford Progeria Syndrome (HGPS), activation of a cryptic splice site within exon 11 of Lamin A by a silent gly-to-gly change at codon 608 (G608G; 150330.0022) leads to the production of an altered form of prelamin A, called progerin: this mutation induces the deletion of 50-amino acids in prelamin A that results in a loss of the endoproteolytic cleavage site; as a consequence, a farnesylated mutant prelamin A, progerin, is formed instead of mature Lamin A and accumulates inside the cells (Coutinho et al., 2009).

In terms of biological function, besides the well-known structural role of lamins, a key action of these proteins in regulation of transcription and epigenetic modifications of chromatin has emerged in the last decade (Dahl et al., 2008). This regulation predominantly occurs at the nuclear periphery, where lamins interact with their binding partners of the inner nuclear membrane and heterochromatin (Gruenbaum et al., 2005); however, Lamin A/C have also been found to be localized in the nucleoplasm, where they exist as a detergent-soluble pool (Kolb et al., 2011) in complex with the lamina-associated polypeptide (LAP) 2α (Dechat et al., 2000) and euchromatic regions (Gesson et al., 2016), and provide a more dynamic and precise regulation of expression of cell-specific and context-dependent genes in response to developmental or environmental cues.

On the other hand, binding of chromatin to the nuclear lamina (NL) occurs at the lamin associated domains (LADs), large genomic regions ranging from 0.1 to 10 megabases (Mb) in size and occupying more than one–third of the genome; LADs are conserved throughout species and mostly characterized by low gene density/expression and markers of heterochromatin (i.e., H3K9me2, H3K9me3, and H3K27me3 histone modifications) (Kind and van Steensel, 2010; Kind et al., 2013; Meuleman et al., 2013). Those regions have been identified and mapped in several species, including *D. melanogaster, C. elegans*, mouse and human cell lines using the adenine methyltransferase technique (DamID) (Pickersgill et al., 2006; Guelen et al., 2008; Ikegami et al., 2010; Peric-Hupkes et al., 2010). These genome-wide studies revealed the existence of two main types of LADs, the constitutive LADs (cLADs), which comprise genomic regions interacting with the nuclear lamina independently by the type of cell, and facultative LADs (fLADs), that are cell-type specific and dynamic through cellular states (i.e., pluripotency vs. differentiation toward specific lineages) (Reddy et al., 2008; Peric-Hupkes et al., 2010; Meuleman et al., 2013). cLADs are more conserved than fLADs amongst species (mouse and human), in terms of size and genomic position, therefore they are proposed to form a structural “backbone” that is crucial to determine the spatial architecture of chromosomes into the nucleus of the cells. Instead, during differentiation, genes involved in development are repositioned toward the nuclear interior, demonstrating the dynamics and flexibility of the interactions within the fLADs. Impairment of this dynamic reorganization of the genome has been reported during terminal differentiation of Emery-Dreifuss Muscular Dystrophy skeletal myoblasts and in human adipose stem cells carrying the lypodystrophy-causing *p*.R482W LMNA mutation (Perovanovic et al., 2016; Oldenburg et al., 2017). However, the understanding of the molecular mechanisms sustaining the interactions of LAD sequences with Lamin A/C is still in its infancy; few evidence are in favor of a link with the chromatin state. The presence of some histone marks at the LAD regions (i.e., H3K9me2/me3; H3K27me3), suggests that G9a, Suv39H1/H2, and EZH2, which are the enzymes catalyzing these modifications, may be direct players in orchestrating NL-genomic loci interaction (Bian et al., 2013; Kind et al., 2013; Chen et al., 2014; Harr et al., 2015) and contribute to finely regulate gene expression: detachment of chromatin from the NL is typically associated to gene activation, while the binding to it generally leads to gene repression (Peric-Hupkes et al., 2010; Robson et al., 2016).

Interestingly, absence of both Lamin A/C and LBR proteins has been described to determine an inversion of the conventional chromatin pattern and to lead to the re-localization of heterochromatin, which is typically localized at the periphery of the nucleus, toward the nuclear interior. In eukaryotes, such inverted architecture has been found in the rod photoreceptor cells of nocturnal mammals, where it serves to reduce light loss from the retina and to allow nocturnal vision (Solovei et al., 2009, 2013). During cell differentiation and development, LBR and Lamin A/C are sequentially expressed in a coordinated fashion to orchestrate the tethering of the heterochromatin to the NL, with LBR expressed in the early stages and then replaced by the appearance of Lamin A/C. However, comparative transcriptional analyses of myoblasts genetically depleted either for LBR or Lamin A/C indicated that the two proteins are not completely interchangeable, as it appears to be in the rod cells, but their action might slightly differ in specific cellular contexts; indeed the two proteins exert opposite effects on a set of muscle genes in the early myogenic cells, but not in those from mature skeletal muscle, supporting a role of the LBR/Lamin A/C expression dynamics in differentiation of a broad range of cells and tissues through regulation of peripheral heterochromatin tethering and transcription (Solovei et al., 2013).

Based on the evidence discussed so far we can conclude that changes in the position of a specific genomic region in the 3D space of the nucleus result in switches amongst transcriptional and cellular states; this supports the concept that spatial genomic architecture is essential for defining distinct cell identities and functions, and that changes of this structural organization is likely to impact cellular pathophysiology.

Besides the LADs, which identified the chromatin distribution with respect to the NL, advances in resolution and sensitivity of the techniques to assess long-range chromatin interactions (i.e., chromatin conformation capture—3C and genome-wide 3C—Hi-C) led to the identification of topological chromatin subdomains, called TADs (namely topologically associated domains), which are units of chromatin in spatial proximity in the nuclear 3D space, mostly comprising regulatory elements, such as enhancers and promoters (Gonzalez-Sandoval and Gasser, 2016). TADs are generally conserved amongst species and cell-types; however, similarly to the LADs, changes in TAD contacts have been reported to occur during differentiation of pluripotent stem cells, supporting the relevance and the cooperation of all these forms of chromatin spatial organization in guiding gene expression in cell fate choices during organ development and cell differentiation (Gonzalez-Sandoval and Gasser, 2016; Krijger et al., 2016; Bonev et al., 2017; Poleshko et al., 2017). A comprehensive review on the hierarchical organization of the 3D genome and the impact on gene regulation and cell fate decisions is given by Bonev and Cavalli (2016).

On this regard, a recent work from Zheng et al. integrated Hi-C and 4C (chromatin conformation capture on chip) conformation studies with fluorescence *in situ* hybridization (FISH), DamID, epigenome, and transcriptome analyses of mouse ESCs depleted for all lamin isoforms and demonstrated how the lamin meshwork overall dictates spatial genomic organization by regulating LADs distribution and controlling TADs interactions (Zheng et al., 2018). Results from this study further reinforce the role of lamins in transcriptional control of pluripotent vs. differentiated states of the cells and in response to environmental cues.

Another important layer of regulation of gene expression in laminopathies can be attributed to the interaction of lamin A to its own binding proteins, that include key epigenetic regulators, such as MAN, SUN 1 and 2, emerin, matrin-3, and proteins of the Polycomb Group (PcG) (Wilson and Foisner, 2010; Cesarini et al., 2015; Depreux et al., 2015). The recent work from Cesarini et al. has shown that loss (or reduction) of Lamin A/C interferes with the myogenic transcriptional program via disassembly and dispersion of PcG proteins (Cesarini et al., 2015); this is in support of an interplay between Lamin A/C and the Polycomb Repressor Complex 2 (PRC2) to achieve temporally regulated gene transcription through development. Consistently, LMNA mutations have also been shown to contribute to biophysical defects of laminopathic cells by altering Lamin A “interactome,” either by disrupting the interaction with specific binding proteins or creating new association pathways; for example, the truncating myopathy-causing Δ303 LMNA mutation disrupts the ability of Lamin A/C to bind to matrin-3, altering the connection of the nuclear lamina with the nucleoplasmic content and the structure of the nuclear lamina itself (Depreux et al., 2015).

All the evidence accumulated so far strongly supports the idea of an epigenetic control laying at the basis of the diverse phenotypes associated to laminopathies, with their heterogeneity resulting from a cell-type specific disruption of the chromatin architecture and the related gene transcription programs.

Indeed, even though Lamins A/C are expressed in almost all differentiated cells, laminopathies manifest with at least 12 different clinical disorders *(*Table 1), mostly presenting with tissue-specific phenotypes, which are often overlapping. These may be grouped into those that affect the striated (cardiac/skeletal) muscle (i.e., Emery-Dreifuss Muscular Dystrophy and Dilated Cardiomyopathy type 1A), the adipose tissue (Familial Partial Lipodystrophy-FPLD and other lypodistrophies, causing metabolic abnormalities) and the peripheral nerves, or give rise to syndromes affecting multiple organs and causing dramatically accelerated aging (i.e., Hutchington-Gilford Progeria Syndrome, atypical Werner Syndrome). A thorough review on the clinical phenotypes of laminopathies is given by Worman and Bonne (2007).

**Table 1:** Diseases caused by LMNA mutations, classified as tissue-specific or systemic-multiorgans disorders.

**Tissue-specific disorders**	**Inheritance**	**Phenotype MIM number**
Cardiomyopathy, Dilated, 1A	AD	115200
Charcot-Marie-Tooth Disorder, Type 2B1	AR	605588
Emery-Dreifuss Muscular Dystrophy 2 (EDMD2)	AD	181350
Emery-Dreifuss Muscular Dystrophy 3 (EDMD3)	AR	616516
Heart-hand syndrome, Slovenian type	AD	610140
Muscular Dystrophy, congenital, LMNA-related	AD	613205
Muscular Dystrophy, Limb-Girdle, Type 1B (LGMD1B)	AD	159001
Malouf Syndrome	AD	212112
Dunnigan-type Familial Partial Lipodystrophy (FPLD)	AD	151660
**Systemic-multiorgans disorders**	**Inheritance**	**Phenotype MIM number**
Hutchinson-Gilford Progeria Syndrome (HGPS)	AD	176670
Restrictive Dermopathy, Lethal	AD	275210
Atypical Werner Syndrome	AD	277700
Mandibular Dysplasia (MAD)	AR	248370
Lipoatrophy with Diabetes, Hepatic Steatosis, Hypertrophic Cardiomyopathy and Leukomalenodermic Papules (LDHCP)	AD	608056

*AD, Autosomal Dominant; AR, Autosomal Recessive; MIM, (Mendelian Inheritance in Man)*.

A “gene regulation” hypothesis was already raised more than a decade ago to explain the occurrence of tissue-specific phenotypes in this group of diseases, and mainly relied on the transcriptional control driven by lamin A on tissue specific genes through its binding (direct or mediated by other proteins) to DNA, histones and transcription factors (Dahl et al., 2008).

Today, thanks to advancements of the technologies for the analysis of DNA sequences and chromatin structure, this view has been expanded and brought to a higher level of complexity, opening new questions on which are the epigenetic regulatory mechanisms occurring in the diverse cell types that are mainly affected by the disease and how these translate into a specific phenotype in the patient.

Abnormal nuclear morphology and genome organization (marker of DNA damage and chromatin modifications) may be considered as common hallmarks of laminopathic cells and have been detected in most of the cells isolated from animal models and patients with different lamin-dependent disorders. Fibroblasts from LMNA null mice, for example, showed altered nuclear envelopes with detached chromatin and mislocalized emerin, indicating that complete loss of Lamin A/C profoundly alters the structure of the inner nuclear membrane and of the nuclear envelope (Sullivan et al., 1999). Following this first study, many others confirmed the effects of Lamin A/C defects on nuclear shape, using either cells depleted for Lamin A/C or carrying point mutations, so that this phenotype is collectively considered a typical sign of a laminopathic cell. Defects in genome organization may be considered a rational consequence of such deep nuclear abnormalities and have been reported in many studies on several patient cell types (i.e., fibroblasts, adipocytes, and skeletal muscle cells). For instance, fibroblasts from HGPS patients exhibit abnormal nuclear morphology associated with loss of heterochromatin markers H3K9me3, HP1α, and HDAC1; also, the presence of progerin in these cells has been associated to impairment of several pathways, including canonical Wnt/β-catenin and TGF-β signaling, and gene expression (Andres and Gonzalez, 2009; Maraldi et al., 2011). Similarly, in cells from EMDM (Emery-Dreifuss Muscular Dystrophy) patients, LMNA mutations perturb the formation of heterochromatin domains leading to pluripotency and cell cycle defects and impairment of myogenesis pathways (Cesarini et al., 2015; Perovanovic et al., 2016). A Lamin A/C-driven epigenetic control of the anti-adipogenic miR-335 locus has been also reported in adipocyte progenitor cells from FPLD2 patients and shown to prevent adipogenic gene expression (Oldenburg et al., 2017).

However, the impact of such genomic reorganization is expected to be highly specific for the diverse cellular contexts and to differently affect the disease phenotypes. As mentioned above, studies so far have been mainly focused on fibroblasts and skeletal muscles cells isolated from patients or obtained from knock-out or transgenic models, while much less information is available on other relevant cell types (such as cardiomyocytes, endothelial cells), which are less accessible or more difficult to culture *in vitro*.

Furthermore, the consequences of LMNA mutations on cell function might be profoundly different, regardless of the type of the mutation, meaning that patients with the same mutation do not necessarily exhibit the same gravity of phenotypes and might have diverse tissue involvement. These observations clearly suggest that cell-specific mechanisms take place, involving cell-specific proteins, genes and chromatin regulators.

Based on these considerations, iPSC technology could represent a useful approach to overcome those limitations and study the mechanisms underlying the occurrence of diverse laminopathy phenotypes and the associated clinical dysfunctions in the proper cellular context. Furthermore, the almost total absence of Lamin A/C expression in pluripotent stem cells and the complete reset of global gene expression in somatic cells after being reprogrammed to pluripotency, render iPSCs a suitable model for developmental studies in laminopathies, which cannot be otherwise investigated in humans.

## iPSC: a General Overview

iPSCs are the result of epigenetic reprogramming of somatic cells (Papp and Plath, 2013). At the initial stages of this process cells are characterized by a general acquisition of dimethylation of lysine 4 on histone H3 (H3K4me2), at the promoter region of “pluripotency” genes, such as Nanog and Oct-4 (Koche et al., 2011). As the reprogramming evolves, changes in dimethylation of lysine 4 on histone H3 (H3K4me2) occurs at the “pluripotency” loci along the genome and determine their active expression that distinguishes the epigenome of a pluripotent cell from the somatic cell of origin (Koche et al., 2011; Fragola et al., 2013). In addition, pluripotent cells (either embryonic stem cells—ESCs or iPSCs) are characterized by a specific epigenetic signature, which is the presence of bivalent trimethylated lysine 4 and 27 on histone H3 (H3K4me3 and H3K27me3) chromatin marks at the promoters of key developmental regulatory genes (Harikumar and Meshorer, 2015). More recent studies brought our knowledge on chromatin architecture organization at a higher level of complexity and revealed that distinct 3D genome structures distinguish somatic cells from those reprogrammed to a pluripotent state and ESCs (Krijger et al., 2016). These diverse genomic organizations are likely to be the results of the remodeling of epigenetic marks, LADs and TAD-TAD interactions occurring during the reprogramming process and after induction of differentiation of iPSCs toward specific cell types (Krijger et al., 2016; Bonev et al., 2017; Poleshko et al., 2017). Altogether, these evidence further support the potential of using pluripotent stem cell-based platforms as a model for investigating epigenetic mechanisms of laminopathies.

iPSCs were first generated by Takahashi and Yamanaka (2006) when they identified a minimum cocktail of transcription factors (TFs) whose overexpression was able to induce acquisition of pluripotency of terminally differentiated mice fibroblasts (Takahashi and Yamanaka, 2006). These TFs (Oct 3/4, Sox2, Nanog, c-Myc) are the key factors that define ESC identity and properties, namely self-renewal and ability to differentiate into derivative of the three germ layers and germ cells (Takahashi and Yamanaka, 2006). iPSCs share these characteristics with ESCs, with the exception of the retainment, in the iPSCs of the epigenetic memory of the cell of origin; so that, the two cell types are considered undistinguishable and both selection and validation of iPSCs are based on testing of the same specific features: expression of pluripotency factors, the ability to differentiate *in vitro* and *in vivo* and, in addition, the verification of the genome and karyotype stability (Kim et al., 2010; Nakahama and Di Pasquale, 2016).

Later in 2007, iPSCs were also obtained from human fibroblasts (Takahashi et al., 2007; Yu et al., 2007) and in the following years many other somatic cell types have been reprogrammed to become pluripotent, with many different strategies that have been improved over time (Malik and Rao, 2013).

After their discovery, iPSCs rapidly became a widely-used tool for disease modeling applications; models of different hereditary and acquired diseases have been generated that successfully recapitulate functional and molecular phenotypes of the diseases in patient-specific relevant cell types, such as neurons for several neurological disorders (Park et al., 2008; Ruan et al., 2012; De Santis et al., 2017), cardiomyocytes for hereditary arrhythmias and cardiomyopathies (Moretti et al., 2010; Sun et al., 2012; Priori et al., 2013; Drawnel et al., 2014; Lodola et al., 2016), skeletal muscle cells for Duchenne Muscular Dystrophy (Li et al., 2015; Long et al., 2018) and endothelial cells for vascular/inflammatory diseases (Adams et al., 2013; Gu et al., 2017).

In addition, recent advancements in genome-editing strategies by site-specific nucleases (such as CRISPR/Cas9 and TALEN) have greatly expanded the possibility to edit the endogenous genome of iPSCs at targeted sites of interest, enabling generation of human cellular models and isogenic lines in which a definite mutation may be investigated with no interference from the patients' genetic background (Hockemeyer and Jaenisch, 2016; Long et al., 2018).

## Cell-specific Insights Into Mechanisms of Laminopathies Through iPSC Technology

The generation of the first human models of “laminopathy” dates back to 2011, with the publications of two studies in which premature aging phenotypes were recapitulated *in vitro* through generation of iPSCs from HGPS patients (Liu et al., 2011a; Zhang et al., 2011). In the same year, additional iPSC lines carrying different LMNA mutations leading to HGPS, atypical Werner Syndrome and dilated cardiomyopathy where also generated and showed that the reprogramming process blunts nuclear morphology abnormalities, no longer detectable in iPSCs, that are then re-acquired when iPSCs are differentiated into secondary fibroblasts (Ho et al., 2011).

These phenomena have been confirmed in almost all the models developed so far, showing normal nuclear morphology of iPSCs derived from any type of laminopathy (Liu et al., 2011b; Chen et al., 2017). These evidences were further strengthened by a study from Ocampo and colleagues, in which induction of partial reprogramming by short term cyclic expression of the “Yamanaka” factors was sufficient to ameliorate cellular and physiological features of premature aging, such as accumulation of DNA damage (increased γ-H2AX foci and expression of age-related stress response genes p16INK4a, p21, and Gadd45b), cellular senescence (MMP13 and IL-6 genes expression), epigenetic defects (loss of heterochromatin marker H3K9me3 and H4K20me3) and nuclear envelope abnormalities, and resulted in the prolongation of the lifespan of HGPS mice (Ocampo et al., 2016).

These reprogramming-induced effects are probably due to the reduction of Lamin A/C levels elicited by the establishment of pluripotency; the role of nuclear lamins in LADs formation also seems to be dispensable in ESCs (Amendola and van Steensel, 2015). Indeed, as we already mentioned earlier in this review, both human ESCs and iPSCs express no or very limited amounts of A-type lamins, whose expression becomes detectable as the differentiation process evolves; similarly, Lamin A/C is absent in early embryos and increases through development (Rober et al., 1989; Constantinescu et al., 2006). A-type lamins seem to be important also in maintenance and differentiation of mesenchymal stem cells (MSCs) and tissue-specific progenitors, by regulating specific gene pathways (Gotzmann and Foisner, 2006). Of note, a role of Lamin A/C on pluripotency potential has been also reported, with high levels of lamins associated with a slower and less efficient induction of iPSCs, most likely because of a negative effect on the expression of pluripotent genes mediated by Lamin A/C (Zuo et al., 2012).

**Figure 1:**
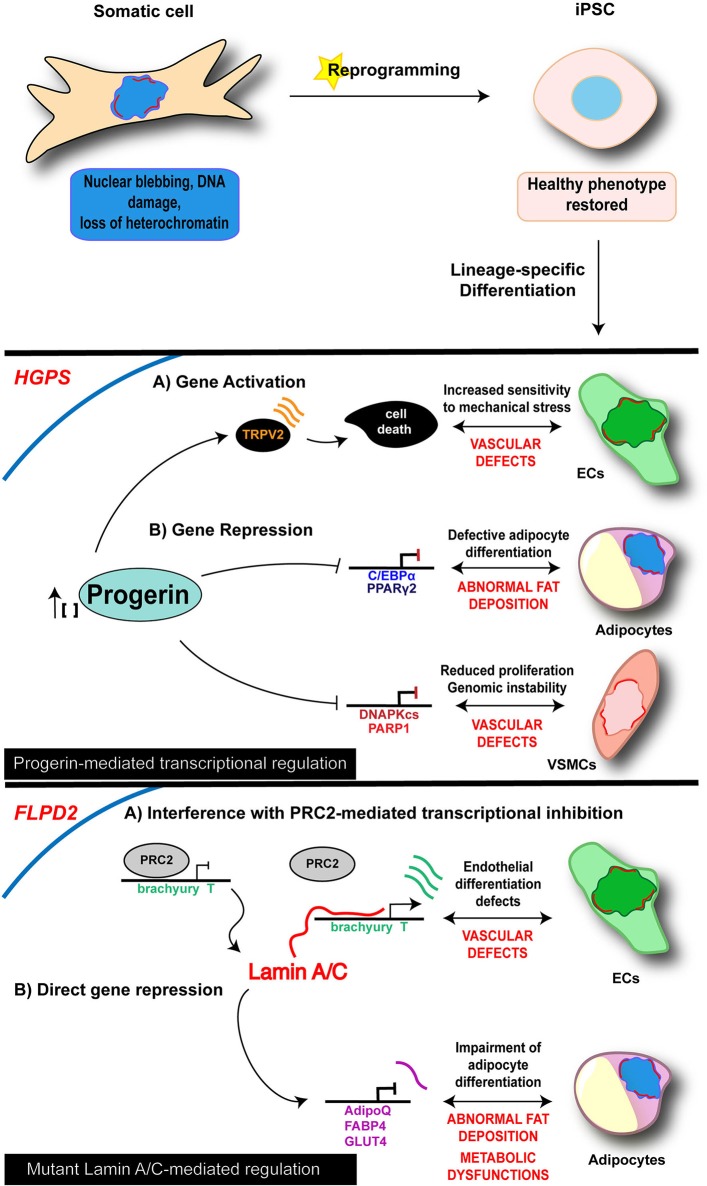
Cell-specific mechanisms of iPSC-derived laminopathy models. Schematic representation of the main mechanisms investigated in iPSC-based models of laminopathies: the proposed pathogenetic mechanisms are presented relative to the specific and disease-relevant cell types. Representation is limited to the diseases and cell types in which an epigenetic involvement has been reported using iPSC technology. Lamin A/C is visualized as a red discontinuous segment in the inner part of the nucleus.

Therefore, reprogramming into iPSCs results in the restoration of the “healthy phenotype” in “laminopathic” somatic cells (Figure 1), and as such may represent the ideal starting point to dissect early molecular mechanisms at the basis of laminopathy and, more generally, of aging processes, allowing to catch the molecular and functional events upon the appearance and accumulation of defective Lamin A/C.

As of today, diverse iPSC lines along with their own disease-relevant differentiated cells (i.e., endothelial cells—ECs; MSCs; vascular smooth muscle cells—VSMCs; cardiomyocytes—CMs) have been developed from 4 different lamin-dependent diseases and carrying 8 different mutations, with most of the studies focused on HGPS. A complete list of models generated so far is provided in the Table 2. Disease-specific findings are discussed in the dedicated sections below and the disease-relevant cell-specific epigenetic mechanisms summarized in the Figure 1.

**Table 2:** iPSC-based models of laminopathies.

**Disease**	**LMNA mutation**	**iPSC-derived cell type**	**Phenotypes**	**References**
HGPS	G608G	MSCs; VSMCs; FBs; ECs; NPs	FBs,VSMCs, MSCs: high progerin levels, DNA damage, nuclear abnormalities VSMCs, MSCs: Reduced viability induced by stress and hypoxia ECs, NPs: no phenotype	Zhang et al., 2011
HGPS	G608G	FBs	Nuclear blebbing, cellular senescence, slow proliferation, electrical induced apoptosis	Ho et al., 2011
HGPS	G608G	VSMCs; FBs	VSMCs, FBs: nuclear lamina disorganization, loss of heterochromatin VSMCs: premature senescence	Liu et al., 2011a
HGPS	G608G	Adipocytes	Severe lipid storage defect at late differentiation stage, PPARγ2 and C/EBPα transcriptional inhibition	Xiong et al., 2013
HGPS	G608G	MSCs	Comparative study evaluating functional effects of the three pharmacological treatments approved for HGPS patients (FTIs, ZoPra, rapamycin)	Blondel et al., 2014
HGPS	G608G	ECs	Cell death due to a sustained [Ca^2+^]_i_ elevation caused by TRPV2 upregulation	Lo et al., 2014
HGPS	G608G	VSMCs	Progerin induced cell death via PARP1 down-regulation	Zhang et al., 2014
HGPS	G608G	MSCs	Identification of mono-aminopyrimidines (Mono-AP) as potential therapeutic molecule for HGPS	Blondel et al., 2016
HGPS	G608G	3D-tissue engineered blood vessels	Recapitulation of HGPS phenotypes in 3D setting, drugs testing	Atchison et al., 2017
HGPS	G608G	iPSCs	Genome-wide analysis of HGPS epigenetic landscape in pluripotent cells	Chen et al., 2017
aWS	E578V	FBs	Nuclear blebbing, cellular senescence, slow proliferation, electrical induced apoptosis	Ho et al., 2011
FPLD2	R482W	Adipocytes	Altered adipogenesis, insulin resistance, increased autophagy	Friesen and Cowan, 2018
FPLD2	R482W	ECs	Defective early vascular differentiation mediated by T/Brachiury deregulation	Briand et al., 2018
DCM	GCCA insertion	FBs	Nuclear blebbing, cellular senescence, slow proliferation, electrical induced apoptosis	Ho et al., 2011
DCM	R225X; GCCA insertion	CMs	Nuclear abnormalities, ERK1/2-mediated susceptibility to electrical stimulation	Siu et al., 2012
DCM	R225X; Q354X; T518fs frameshift mutation	CMs	R225X-CMs: Improved senescence, apoptosis, excitation-contraction coupling, and contractile functions induced by PTC124 treatment	Lee et al., 2017
DCM	R190W	CMs	Sarcomeric disorganization, abnormal activation of ERK1/2 signaling	Chatzifrangkeskou et al., 2018

*List of the reported models of laminopathy-related diseases generated through iPSC technology and schematic description of the phenotypes detected in the diverse analyzed cell types. MSCs, mesenchymal stem cells; VSMCs, vascular smooth muscle cells; FBs, fibroblasts; ECs, endothelial cells; NPs, neural progenitors; CMs, cardiomyocytes*.

### Premature Aging and Progeria

Studies on HGPS-iPSC models were the first to appear and are undoubtedly the most advanced in the field: different groups have contributed in dissecting the diverse tissue-specific phenotypes in the last years, from vascular defects to adipose tissue formation, mesenchymal, and smooth muscle cells biology (Liu et al., 2011a; Misteli, 2011; Zhang et al., 2011, 2014; Xiong et al., 2013; Lo et al., 2014; Atchison et al., 2017; Chen et al., 2017).

In 2011, Zhang et al. reported the generation of iPSCs from HGPS dermal fibroblasts and analyzed the impact of progerin accumulation in cells of both mesenchymal and non-mesenchymal derivation (Zhang et al., 2011). Their study revealed that the various cell types differentiated from iPSC lines express different amounts of progerin, which was higher in MSCs, VSMCs and fibroblasts, while very low levels of the protein were detectable in neural progenitors (NPs). Consistently, MSCs, VSMCs and fibroblasts were severely affected by increased DNA damage, the typical nuclear abnormalities and increased sensitivity to hypoxia and stress by electrical stimulation; those phenotypic traits were instead absent in NP cells. In a subsequent study, a loss of proliferative potential of VSCMs was recorded and attributed to a downregulation of the poly (ADP-ribose) polymerase 1 (PARP-1), also leading to an increase in the chromosomal aberration in those cells through the activation of the non-homologous joining pathway (Zhang et al., 2014). Accumulation of progerin has been associated to gene expression changes also in other HGPS-iPSC models, providing more hints on the molecular basis of the diseases. Liu *et al* showed an association of progerin accumulation with the repression of DNA-dependent protein kinase catalytic subunit (DNAPKcs) gene, whose loss leads to an impairment of the proliferative potential of VSCMs (Liu et al., 2011a). On the other hand, induction of expression of the vanilloid transient receptor potential cation channel 2 (TRPV2) upon mechanical stimulation has been recorded in iPSC-derived HGPS-ECs and is thought to be responsible for the increased sensitivity to mechanical stress in these cells, through the induction of Ca^2+^ overload and apoptosis (Lo et al., 2014).

Presence of DNA damage was also a common feature in VSMCs from HGSP-iPSCs, and was reported by two additional studies, in association with heterochromatin defects, nuclear disorganization and premature senescence (Liu et al., 2011a; Chen et al., 2017).

Interestingly, reorganization of nuclear architecture and remodeling of epigenetic landscape occur during the transition from HGPS fibroblasts to iPSCs and then through lineage-specific differentiation of HGPS-iPSCs, and may be considered as the “*primum movens*” behind the functional and phenotypic defects of HGPS cells. Indeed, epigenomic assessment of gene methylation, transcription and heterochromatin markers (H3K9me3 and HP1α) revealed that control and HGPS pluripotent lines are much more similar to each other than the parental fibroblasts, and that this similarity is lost with differentiation, resulting in the appearance of cellular defects, such as nuclear abnormalities and senescence (Liu et al., 2011a). This view is further corroborated by genome-wide and structural epigenetic studies, that demonstrated a rescue of the global mRNA profile along with the distribution of H3K4me3 and H3K27me3 at proximal promoters and chromatin conformation in HGPS-iPSCs (Chen et al., 2017). Interestingly, the authors showed that HGPS-iPSCs can differentiate into VSMCs that, in an initial phase, do not exhibit any phenotype, thus providing an *in vitro* model in which initiation and progression of the molecular phenotypes can be monitored over time.

Loss of adipose tissue is another typical feature of HGPS, as well as of the other LMNA-dependent lipodystrophic syndromes; affected patients present complete or partial fat atrophy (lipoatrophy) and metabolic defects (such as insulin resistance). Impairment of the adipocyte formation process is thought to be at the basis of these clinical manifestations. Results from Xiong et al in iPSC-derived adipocytes differentiated from HGPS-iPSC lines go toward this direction and support a role of Lamin A/C in adipocyte differentiation through the regulation of PPARγ2 and C/EBPα (Xiong et al., 2013). Moreover, they also showed that progerin interferes with late adipogenic regulators, by controlling expression of “maturation” genes during the terminal phase of differentiation; this late-stage control is probably responsible for the metabolic dysfunctions associated to the disease.

Finally, in the context of premature aging, iPSCs have also been generated from a patient with atypical Werner Syndrome (aWS), carrying the LMNA-E578V mutation (Ho et al., 2011). A-WS shares some of the clinical features with HGPS, such as lipodystrophy, atherosclerosis, thinning/graying hair, and skin atrophy (Mounkes and Stewart, 2004). Similarly, a-WS fibroblasts exhibit cellular features common to HGPS cells, as abnormal nuclear morphology, cellular senescence and chromosomal instability, that are all reset by reprogramming to iPSCs and re-acquired upon re-differentiation into secondary fibroblasts (Ho et al., 2011). However, investigations are still in their infancy and whole-genome studies at multiple levels are needed in order to get a comprehensive view of the pathogenetic mechanisms underlying the disease in multiple affected tissues.

Nonetheless, some studies already move forward in the field and used iPSC-models, in particular those generated from HGPS, for preclinical comparative pharmacological tests and for evaluation of side effect of therapies that are currently undergoing clinical trials (Blondel et al., 2014, 2016; Atchison et al., 2017). This topic is further discussed in the next section.

### Lipodystrophies

Lipodystrophies are rare diseases, mainly characterized by loss of adipose tissue, accompanied by metabolic dysfunctions induced by ectopic fat deposition. Defects in adipocyte differentiation, triglyceride synthesis, lipolysis, and lipid droplet structure or biogenesis are the proposed pathophysiological mechanisms. LMNA mutations are the cause of the most frequent genetic form of lipodystrophy, whose typical form is the Familial Partial Lipodystrophy of the Dunnigan type (FPLD2), mainly due to the *p*.R482W mutation in the C-terminal domain of Lamin A (Vigouroux et al., 2018)

Two FLDP2-iPSC models have been generated so far, and their investigation has contributed to advance our knowledge on the pathophysiological mechanisms in two distinct cellular contexts: the adipocyte, the main component of the adipose tissue (Friesen and Cowan, 2018), and the endothelial cell (EC), one of the cell-types of the vascular compartment (Briand et al., 2018). Indeed, early onset of atherosclerosis often manifests in FPLD2 patients, causing premature occurrence of cardiovascular events, such as coronary heart disease, peripheral arteritis and stroke.

A common hallmark that emerged from these studies is an effect of *p*.R482W Lamin A/C mutation on developmental potential of FPLD2-iPSCs, leading to decreased adipocyte differentiation efficiency and to a deregulation of EC induction. In both cases the events are driven by an alteration of the gene expression program through cell fate induction (Briand et al., 2018; Friesen and Cowan, 2018).

More in detail, in the study from Frieses and Cowan, the impairment of the differentiation efficiency into adipocytes was coupled with a reduced expression of late adipocytes markers (AdipoQ, FABP4, GLUT4), leading to functional defects including insulin resistance, increased lipolysis and a metabolic switch toward fatty acid oxidation (Friesen and Cowan, 2018).

Premature and prolonged expression of the mesodermal transcription factor Brachiury T-box was instead at the basis of the endothelial differentiation defects reported by Briand and colleagues. The proposed mechanism relies on the disruption of the PRC2-dependent repressor block at the T/Brachiury locus mediated by the mutated Lamin A/C and resulting in the spatial repositioning of the mesodermal locus toward the nuclear interior. The described molecular events were completely rescued in ECs differentiated from gene-edited wild type iPSC lines, further reinforcing the view of a key role of Lamin A/C on orchestrating chromatin architecture (Briand et al., 2018).

### Dilated Cardiomyopathy

Only few iPSC models of lamin-dependent dilated cardiomyopathy (DCM) have been reported so far and mostly regard nonsense mutations (R225X, GCCA insertion, Q354X, and T518 frameshift), thus acting through a haploinsufficiency mechanism.

Studies conducted up to now are mostly phenomenological, and were able to recapitulate some morphological/functional defects typical of laminopathic cells in either secondary (iPSC-derived) fibroblasts (Ho et al., 2011) or cardiomyocytes (CMs) (Siu et al., 2012; Lee et al., 2017), while no investigations have been conducted to underpin epigenetic regulation mechanisms in cardiac-relevant cells.

Cellular abnormalities detected in CMs differentiated from all iPSC lines are akin to those already described in cells of different derivation, including nuclear abnormalities, senescence, apoptosis, and susceptibility to electrical stress. In particular, Siu et al. showed that electrical stimulation exacerbates phenotypic defects in R225X and frameshift CMs, through an ERK $\frac{1}{2}$ dependent mechanism (Siu et al., 2012). More recently, abnormal activation of ERK1/2 signaling was also reported in iPSC-CMs carrying the R190W *LMNA* mutation and was associated to defects in the sarcomere organization (Chatzifrangkeskou et al., 2018).

On the other hand, no studies so far have investigated the epigenetic role of Lamin A/C and their mutations in cardiac cells. However, we can speculate that epigenetic mechanisms similar to those described in other cell types could also take place in a cell-specific fashion in CMs, and be at the basis of cardiac abnormalities of patients with LMNA-dependent cardiomyopathy. On this regard, our group has accumulated evidence that a defective epigenetic regulation mediated by Lamin A/C may be at the basis of CM dysfunctions, through the perturbation of expression of genes encoding proteins involved in the main functional processes of the CMs [Abstract:(Crasto et al., 2017)].

Further investigations aiming at unveiling this layer of regulation are highly desirable and are of utmost importance for the development of more effective therapies.

Apropos to this, a recent study showed that typical cellular phenotypes of DCM-CMs were abrogated by treatment with ataluren (PTC124). This small molecule has been approved by the FDA for treatment of genetic diseases due to non-sense mutations, and functions by over-riding premature stop codons (Lee et al., 2017). An improvement of the excitation-contraction coupling was also recorded in mutant CMs following drug administration *in vitro*.

## Translational Potential of iPSC Models of Laminopathies

Drug testing is a major application of iPSC technology (Takahashi and Yamanaka, 2016). In the last few years, iPSC-derived cells have been employed as a platform to test for the efficacy of drugs, for toxicity screening and predictive pharmacology, and to search for new therapeutic molecules (Del Alamo et al., 2016; Cayo et al., 2017; Kondo et al., 2017). These studies demonstrated the feasibility of the approach, as long as a reliable readout can be used (Elitt et al., 2018).

Few studies testing the efficacy of pharmacological therapies using iPSC-derived models of laminopathies have been recently published and reported the effects of drugs currently in clinical trials or approved for patients' use on functional and molecular parameters in iPSC-derived cells (Blondel et al., 2014, 2016; Atchison et al., 2017; Lee et al., 2017). In the first study, a systematic comparative analysis of the three main treatments for HGPS (the farnesyltransferases inhibitor lonafarnib, the combination of pravastatin and zoledronate, and rapamycin) have been carried out in iPSC-MSCs, using nuclear morphology, progerin accumulation and prelamin A maturation (the main HGPS cellular traits) as readouts. Consequences of the three pharmacological treatments have been also evaluated on secondary functional parameters such as cell proliferation and osteogenic differentiation (Blondel et al., 2014). Beyond the demonstration of the efficacy of all treatments on rescue of the nuclear shape defects, the three tested drugs displayed differences in their therapeutic potential and exerted diverse effects on cell functional parameters, that have not been reported before, although already used for patients' treatment.

In another study, Atchison et al. recapitulated the complexity of blood vessels in an organoid-like 3D tissue-engineered model made up of VSMCs and endothelial progenitor cells and demonstrated that treatment with Everolimus (an mTOR inhibitor) was able to improve vasoactivity and VSCM differentiation (Atchison et al., 2017).

As mentioned above, iPSC-derived CMs carrying the R225X LMNA mutation have been also successfully employed as a platform to investigate the effect of another drug, PTC124 (ataluren), that acts by promoting read-through of premature stop codons, and has already been applied to patients with Duchenne Muscular Dystrophy. In that study, the authors observed both a reduction of morphological defects at the nuclear level and an improvement of the functional performance of mutated cardiac cells (Lee et al., 2017).

On the whole, results from these studies demonstrate that it is feasible to use iPSCs for preventive testing of the efficacy of drugs in disease-relevant cell types by scoring diverse cellular and molecular targets; not least, these data also provide a solid ground to move toward high-throughput-based testing level to search for new molecules with a therapeutic value.

The second study from Blondel et al. describes the identification of monoaminopyrimidines, a new family of inhibitors of farnesylation, through the screening of 21608 small molecules on iPSC-MSCs (Blondel et al., 2016).

Furthermore, iPSC models may be of particular relevance for assessing the pharmacological value of molecules emerged from recent studies for having a beneficial effect on HGPS hallmarks, *in vitro*. Such molecules include metformin (Egesipe et al., 2016), methylene blue (Xiong et al., 2016), and remodelin (Larrieu et al., 2014; Balmus et al., 2018), and have great potential to be quickly translated to clinical use.

Besides the usefulness of iPSC-models in testing drug efficacy, these are also a powerful tool to assess their side effects at diverse cell and tissue levels; this possibility is extremely relevant to facilitate their clinical use in the context of rare diseases, for which design and execution of informative clinical trials is particularly challenging.

## Concluding Remarks

IPSCs offer the possibility to investigate pathogenetic mechanisms of laminopathies at levels that were unthinkable only a decade ago. Starting from a somatic cell of a patient (either fibroblasts or peripheral blood mononuclear cells), cell types of any derivation may be obtained and allow to investigate a human disease in the relevant cellular context or organ-like 3D structures (organoids) (Orqueda et al., 2016; Takahashi and Yamanaka, 2016). This is of key importance for laminopathies, in which several districts of the body and interacting cells types are simultaneously affected.

Given the important role of Lamin A/C as regulators of chromatin architecture and gene expression, coupled with the developmental- and cell-specific nature of such epigenetic regulations, the use of iPSC-derived models is crucial to pursue gaining knowledge on the pathophysiological mechanisms at the basis of LMNA-related diseases.

In addition, iPSCs represent the solely available strategy to dissect the role of Lamin A/C in human development and lineage commitment/specification, which have been shown to be at the basis of many functional abnormalities of the disease.

One of the major advantages of iPSCs lies in the possibility of a rapid and direct translation of the obtained findings for the patients' benefit (Li et al., 2017): many cell lines have been generated so far in different laboratories worldwide, and many others will come, that have served to identify patient-specific cellular and molecular traits. These advancements are fundamental to pinpoint new biological readouts for pharmacological screening, toxicity tests and drug development applications.

With the availability of optimized differentiation protocols and the improvement of technological supports, we envision application of “personalized” models for preventive screening to assess patient's drug response and development of a “personalized” therapeutic strategy that suits best on any patient, in the future.

## Author Contributions

SC performed the bibliographic search and contributed to writing the manuscript. EDP wrote and revised the manuscript, provided funding.

### Conflict of Interest Statement

The authors declare that the research was conducted in the absence of any commercial or financial relationships that could be construed as a potential conflict of interest.
